# BioFire FilmArray Pneumonia Panel enhances detection of pathogens and antimicrobial resistance in lower respiratory tract specimens

**DOI:** 10.1186/s12941-022-00512-8

**Published:** 2022-06-04

**Authors:** Kosuke Kosai, Norihiko Akamatsu, Kenji Ota, Fujiko Mitsumoto-Kaseida, Kei Sakamoto, Hiroo Hasegawa, Koichi Izumikawa, Hiroshi Mukae, Katsunori Yanagihara

**Affiliations:** 1 Department of Laboratory Medicine, Nagasaki University Hospital, 1-7-1 Sakamoto, Nagasaki, Nagasaki 852-8501 Japan; 2 Department of Laboratory Medicine, Nagasaki University Graduate School of Biomedical Sciences, Nagasaki, Japan; 3 Department of Infectious Diseases, Nagasaki University Graduate School of Biomedical Sciences, Nagasaki, Japan; 4 Department of Respiratory Medicine, Nagasaki University Graduate School of Biomedical Sciences, Nagasaki, Japan

**Keywords:** Lower respiratory tract infections, Multiplex molecular assay, Automated system, Rapid detection

## Abstract

**Background:**

This study investigated the diagnostic utility of the BioFire FilmArray Pneumonia Panel (PN panel), an automated and multiplexed nucleic acid detection system that rapidly detects 26 pathogens (18 bacteria and eight viruses) and seven antimicrobial resistance markers in a single assay.

**Methods:**

We analyzed the targets in lower respiratory tract specimens using the PN panel and compared the detection results with those of bacterial culture methods and antimicrobial susceptibility testing.

**Results:**

Of the 57 samples analyzed, the PN panel detected 97 targets (84 bacteria, four viruses, and nine antimicrobial resistance markers). Detection of bacteria and antimicrobial resistance was three times greater than that of the bacterial culture (25 bacteria and two resistant isolates) against the targets available in the panel. The overall positive and negative percent agreements between the PN panel and culture methods for bacterial detection were 100.0% and 92.9%, respectively. Multiple pathogens were detected by the PN panel in 24 samples (42.1%), ranging from two pathogens in 11 samples (19.3%) to six pathogens in one sample (1.8%). The PN panel semiquantitatively detected higher copies (≥ 10^6^ copies/mL) of bacterial targets if the bacteria were positive by the culture method. In contrast, the semiquantitative values obtained by the panel varied (10^4^ to 10^7^ ≤ copies/mL) among bacteria that were negative by the culture method.

**Conclusions:**

The PN panel enhanced the detection of pathogens and antimicrobial resistance markers in lower respiratory tract specimens.

## Background

Lower respiratory tract infections (LRIs) are a significant public health concern and a leading cause of death from infection worldwide [1]. Therapeutic strategies for LRIs, mainly antimicrobial selection, consist of empirical and definitive therapies [2–4]. Initial empirical therapy covers the major causative pathogens that are predicted, while antimicrobials for definitive therapy are selected against identified pathogens in consideration of antimicrobial susceptibility. Several guidelines and studies have reported the isolation frequency of pathogens and the risk factors for the acquisition of antibiotic-resistant bacteria [3–5]. However, it may be difficult to accurately predict etiological agents at the time of empiric therapy because of differences in regional epidemiology and medical settings. Additionally, culture-based bacterial identification and antimicrobial susceptibility testing (AST) are useful for definitive therapy but are time intensive.

The FilmArray system is an automated and multiplexed nucleic acid detection system, and the BioFire FilmArray Pneumonia Panel (PN panel) simultaneously detects 33 targets (26 pathogens and seven antimicrobial resistance markers) in approximately 75 min in a simple assay procedure.

In this study, we analyzed these targets in lower respiratory tract specimens using the PN panel and compared the results with those of bacterial culture and AST.

## Methods

### Study design and samples

Sputum, tracheal aspirates, bronchoalveolar lavage fluid (BAL), and mini-BAL specimens, which were submitted to the clinical laboratory for microbiological evaluation, were collected at the Nagasaki University Hospital between October 2020 and January 2021. The hospital had 874 beds, and the average number of inpatients and outpatients were 669 and 1653 patients per day, respectively, during this period. The quality of sputum and tracheal aspirates was assessed according to the Miller and Jones classification [6]. Samples were randomly selected from specimens P1 (purulent, grade 1—pus amounting to less than one-third of the specimen), P2 (purulent, grade 2—pus amounting to one-third to two-thirds of the specimen), and P3 (purulent, grade 3—pus amounting to more than two-thirds of the specimen). De-duplication was performed if samples were repeatedly collected from a single patient, and samples from unique patients were included in this study. The specimens were stored at − 80 °C until assay using the PN panel.

### Culture methods

Sputum and tracheal aspirate samples, which were prepared using Sputazyme (Kyokuto Pharmaceutical Industrial Co., Ltd.), and BAL and mini-BAL samples were streaked using 10 µL loop and cultured on Nissui Separated Plate Sheep Blood Agar/Chocolate Agar EXII (NISSUI PHARMACEUTICAL CO., LTD.), CHROMagar Candida/BTB agar (Prepared media) (KANTO CHEMICAL CO., INC.). Additionally, if the physician specifically requested testing for methicillin-resistant *Staphylococcus aureus* (MRSA), the samples were cultured on Pourmedia MRSA SELECTIVE AGAR II (Eiken Chemical Co., Ltd.). All bacteria that were considered pathogens, excluding normal flora, were picked up and identified even if they were cultured in rare or few quantities. After pure culture, isolates were identified using matrix-assisted laser desorption ionization time-of-flight mass spectrometry (MALDI-TOF MS), MALDI Biotyper system (Bruker Daltonik, GmbH). *Streptococcus pneumoniae* was identified using the optochin disc diffusion test.

Antimicrobial susceptibility was determined using a BD Phoenix M50 (Becton Dickinson). MRSA and extended-spectrum β-lactamase (ESBL) producers were automatically determined using BD Phoenix M50. Antimicrobial susceptibility of *Haemophilus influenzae* was determined using minimal inhibitory concentration (MIC) plates customized by Eiken Chemical Co., Ltd.

### Analysis using the BioFire FilmArray Pneumonia Panel

The BioFire FilmArray Pneumonia Panel (bioMérieux Japan Ltd.), which has been approved by Food and Drug Administration for use in clinical settings in the United States, simultaneously detects 18 bacterial targets (*Acinetobacter calcoaceticus-baumannii* complex, *Enterobacter cloacae* complex, *Escherichia coli*, *H. influenzae*, *Klebsiella aerogenes*, *Klebsiella oxytoca*, *Klebsiella pneumoniae* group, *Moraxella catarrhalis*, *Proteus* species, *Pseudomonas aeruginosa*, *Serratia marcescens*, *S. aureus*, *Streptococcus agalactiae*, *S. pneumoniae*, *Streptococcus pyogenes*, *Chlamydia pneumoniae*, *Legionella pneumophila*, and *Mycoplasma pneumoniae*), eight viral targets (adenovirus, coronavirus, human metapneumovirus, human rhinovirus/enterovirus, influenza A, influenza B, parainfluenza virus, and respiratory syncytial virus), and seven targets related to antimicrobial resistance (*mecA*/*mecC* and staphylococcal cassette chromosome *mec* [SCC*mec*] right-extremity junction [MREJ], KPC, NDM, OXA-48-like, VIM, IMP, and CTX-M) on the FilmArray system.

The BioFire FilmArray Pneumonia Panel pouch, which is a closed and disposable system, contains all the reagents required for nucleic acid extraction and purification, reverse transcription, and PCR. The stored specimens were analyzed using the PN panel. Briefly, the pouch was hydrated by the hydration injection vial via the pouch hydration port. Approximately 200, 50, or 10 µL of the specimen was mixed with the sample buffer using a vortex mixer. After flashing in a centrifuge to remove foam, the mixture was transferred to the sample injection vial and injected into the pouch via the pouch sample port. The sample preparation process, including the sample volume used for the assay, was off-label. The prepared pouch was inserted into the instrument, and subsequent assay steps (nucleic acid extraction and purification, nested multiplex PCR, and detection of each target in the array) were automatically performed on the system. The system reports the detection results of pathogen and antimicrobial resistance markers and the semiquantification results of the detected bacterial targets excluding three atypical bacteria.

### Comparison between the PN panel and bacterial culture-based methods

The detection results of the PN panel were compared with those of bacterial culture methods and AST, and positive and negative percent agreements (PPA and NPA) were calculated as follows: PPA, the number of concordant positive results divided by the number of all positive results by culture-based methods; NPA, the number of concordant negative results divided by the number of all negative results by culture-based methods. The 95% confidence intervals were calculated using R (version 3.5.2) [7].

## Results

A total of 57 specimens (35 sputum, nine tracheal aspirate, and 13 BAL or mini-BAL specimens) were included in this study. Of the 44 sputum and tracheal aspirate specimens, one, 13, and 30 were identified as P1, P2, and P3, respectively, according to the Miller and Jones classification. The average patient age was 66.7 years, and 36 patients (63.2%) were male.

The frequencies of the detected bacteria and antimicrobial resistance are presented in Table 1. The PN panel detected 84 bacteria and nine antimicrobial resistance markers. The panel detected *P. aeruginosa* and *S. aureus* (17 targets each) most frequently, followed by the 10 *K. pneumoniae* group, six *A. calcoaceticus-baumannii* complex, six *E. cloacae* complex, and six *H. influenzae*. With respect to antimicrobial resistance markers, the PN panel detected five *mecA*/*mecC* and MREJ genes, as well as four CTX-M genes.

**Table 1:** Bacteria and antimicrobial resistance identified using the BioFire FilmArray Pneumonia Panel and culture-based methods

Bacterium and antimicrobial resistance^a^	BioFire FilmArray Pneumonia Panel^b^	Culture-based methods^c^
Bacterium		
*Acinetobacter calcoaceticus-baumannii* complex	6	0
*Enterobacter cloacae* complex	6	1
*Escherichia coli*	4	1
*Haemophilus influenzae*	6	5
*Klebsiella aerogenes*	4	0
*Klebsiella oxytoca*	1	1
*Klebsiella pneumoniae* group	10	3^d^
*Moraxella catarrhalis*	2	0
*Pseudomonas aeruginosa*	17	8
*Serratia marcescens*	3	1
*Staphylococcus aureus*	17	3
*Streptococcus agalactiae*	3	0
*Streptococcus pneumoniae*	4	2
*Streptococcus pyogenes*	1	0
Total	84	25
Antimicrobial resistance		
*mecA*/*mecC* and MREJ	5^e^	0^f^
CTX-M	4^g^	2^h^
Total	9	2

^a^Targets available in the BioFire FilmArray Pneumonia Panel

^b^Sample preparation process, including the sample volume used for the assay, was off-label

^c^Culture methods and antimicrobial susceptibility testing

^d^Two and one were identified as *K. pneumoniae* and *K. variicola*, respectively

^e^Detected with *S. aureus*

^f^No methicillin-resistant *Staphylococcus aureus* was detected using culture-based methods

^g^Two were detected with *K. pneumoniae* group and two were detected with both *K. pneumoniae* group and *E. coli*

^h^One *K. pneumoniae* and one *E. coli* were detected as ESBL producers

Meanwhile, 25 bacteria were isolated using culture methods, with *P. aeruginosa* (eight isolates) isolated most frequently, followed by five *H. influenzae*, three strains in *K. pneumoniae* group, and three *S. aureus*. Two ESBL producers were isolated based on their AST results.

The PN panel detected four rhinovirus/enterovirus targets, that were not tested using the reference method in this study.

Table 2 shows the concordance of the detection results for bacteria between the PN panel and culture methods. The PN panel correctly detected all bacterial targets isolated by the culture methods if bacteria were included in the targets of the panel, indicating that the PPA was 100%. The NPAs ranged from 74.1 to 100% and the overall NPA for bacterial detection was 92.9%. *Enterococcus faecalis*, *Enterococcus faecium*, *Stenotrophomonas maltophilia*, *Corynebacterium propinquum*, and *Candida albicans*, which were not targeted by the PN panel, were isolated from one sample each using culture methods. The overall PPA and NPA for the detection of MRSA and ESBL producers were 100.0% and 93.8%, respectively.

**Table 2:** Comparison of detection results against bacteria and antimicrobial resistance between the BioFire FilmArray Pneumonia Panel^a^ and culture-based methods

Bacterium and antimicrobial resistance	PN+, CM+	PN+, CM−	PN−, CM+	PN−, CM−	PPA (95% CI)	NPA (95% CI)
Bacterium						
*A. calcoaceticus-baumannii* complex	0	6	0	51	–	89.5 (78.5–96.0)
*E. cloacae* complex	1	5	0	51	100.0 (2.5–100.0)	91.1 (80.4–97.0)
*E. coli*	1	3	0	53	100.0 (2.5–100.0)	94.6 (85.1–98.9)
*H. influenzae*	5	1	0	51	100.0 (47.8–100.0)	98.1 (89.7–100.0)
*K. aerogenes*	0	4	0	53	–	93.0 (83.0–98.1)
*K. oxytoca*	1	0	0	56	100.0 (2.5–100.0)	100.0 (93.6–100.0)
*K. pneumoniae* group	3	7	0	47	100.0 (29.2–100.0)	87.0 (75.1–94.6)
*M. catarrhalis*	0	2	0	55	–	96.5 (87.9–99.6)
*Proteus* species	0	0	0	57	–	100.0 (93.7–100.0)
*P. aeruginosa*	8	9	0	40	100.0 (63.1–100.0)	81.6 (68.0–91.2)
*S. marcescens*	1	2	0	54	100.0 (2.5–100.0)	96.4 (87.7–99.6)
*S. aureus*	3	14	0	40	100.0 (29.2–100.0)	74.1 (60.3–85.0)
*S. agalactiae*	0	3	0	54	–	94.7 (85.4–98.9)
*S. pneumoniae*	2	2	0	53	100.0 (15.8–100.0)	96.4 (87.5–99.6)
*S. pyogenes*	0	1	0	56	–	98.2 (90.6–100.0)
Total	25	59	0	771	100.0 (86.3–100.0)	92.9 (90.9–94.5)
Antimicrobial resistance						
MRSA^b^	0	5	0	52	–	91.2 (80.7–97.1)
ESBL producer^c^	2	2	0	53	100.0 (15.8–100.0)	96.4 (87.5–99.6)
Total	2	7	0	105	100.0 (15.8–100.0)	93.8 (87.5–97.5)

*PN* BioFire FilmArray Pneumonia Panel, *CM* culture-based methods (culture methods and antimicrobial susceptibility testing), *MRSA* methicillin-resistant *Staphylococcus aureus*, *ESBL* extended-spectrum β-lactamase, *PPA* positive percent agreement, *NPA* negative percent agreement, *CI* confidence interval

^a^Sample preparation process, including the sample volume used for the assay, was off-label

^b^MRSA was detected as *mecA*/*mecC* and MREJ genes using the BioFire FilmArray Pneumonia Panel

^c^ESBL producers were detected as CTX-M genes using the BioFire FilmArray Pneumonia Panel

Table 3 presents the number of samples in which multiple pathogens were detected by the PN panel and culture methods. Of the 57 samples included in the study, multiple pathogens were detected in 24 samples (42.1%) using the PN panel, ranging from two pathogens in 11 samples (19.3%) to six pathogens in one sample (1.8%). Conversely, the culture method detected two pathogens in four samples (7.0%).

**Table 3:** Number of samples in which multiple pathogens were detected using the BioFire FilmArray Pneumonia Panel and culture methods

BioFire FilmArray Pneumonia Panel^a^	Culture methods			Total
	0	1	2	
0	16	1	0	17
1	10	6	0	16
2	2	7	2	11
3	1	4	1	6
4	0	3	1	4
5	1	1	0	2
6	1	0	0	1
Total	31	22	4	57

Detected pathogens were included, even though they were not available in the BioFire FilmArray Pneumonia Panel

^a^Sample preparation process, including the sample volume used for the assay, was off-label

Table 4 summarizes the semiquantitative values of the bacteria measured using the PN panel. The PN panel detected bacterial targets of 10^6^ copies/mL or greater if the bacteria were positive by the culture method. Conversely, semiquantitative values determined by the panel ranged from ≥ 10^7^ copies/mL (13 bacteria) to 10^4^ copies/mL (24 bacteria) in culture-negative samples.

**Table 4:** Semiquantitative values of bacteria measured by the BioFire FilmArray Pneumonia Panel in culture-positive and culture-negative samples

BioFire FilmArray Pneumonia Panel (copies/mL)^a^	Culture methods		Total
	Positive	Negative	
≥ 10^7^	23	13	36
10^6^	2	10	12
10^5^	0	12	12
10^4^	0	24	24
Total	25	59	84

^a^Sample preparation process, including the sample volume used for the assay, was off-label

## Discussion

Our study demonstrated that the PN panel rapidly and effectively detected a variety of pathogens in the lower respiratory tract specimens. The PN panel detected 97 targets (84 bacteria, four viruses, and nine antimicrobial resistance markers), and the detection of bacteria and antimicrobial resistance was three times greater than that of the culture-based methods (25 bacteria and two resistant isolates) (Table 1). Several previous studies have shown similar results in that the panel detected more bacterial targets than the culture method [8, 9]. A recent study reported that the PN panel identified nearly twice as many total bacterial targets as standard-of-care culture in BAL specimens [8]. Another study reported that the PN panel detected one or more bacterial targets in an additional 20% of patients compared to culture methods [10]. In addition, our results also showed that the PN panel correctly detected all bacteria that were detected by the culture methods (PPA, 100%) if they were included in the targets of the panel (Table 2), and that the overall NPA for bacterial detection was 92.9%. Similarly, previous studies reported high performance of the PN panel for bacterial detection with PPAs of 90.0–98.4% and NPAs of 96.8–98.1% [8, 9, 11]. In this study, only four bacterial species (*E. faecalis*, *E. faecium*, *S. maltophilia, C. propinquum*) and one *C. albicans*, which were unavailable in the PN panel, were isolated using the culture method. A recent study from Taiwan reported that the PN panel may cover approximately 70–90% of the most prevalent pathogens causing moderate to severe community-acquired pneumonia in adults, as well as 70–80% of those causing healthcare-associated pneumonia [9]. The current diagnostic strategies require multiple tests to detect possible pathogens. Bacterial culture is the gold standard for bacterial detection, but it is time consuming. The PN panel, which can simultaneously detect major pathogens in a short time, can be useful for the diagnosis of LRIs.

Although atypical bacteria were not detected by the PN panel in this study, recent studies have reported that the PN panel detected bacteria such as *L. pneumophila* and *M. pneumonia*e in a few samples [9, 12].

We found four samples in which rhinovirus/enterovirus was simultaneously detected with the bacteria. Previous studies have reported that co-infection with viruses and bacteria contributes to increased disease severity and mortality [13, 14]. Although we could not assess the relationship between co-infection and disease severity, comprehensive pathogen detection by the PN panel might contribute to elucidating the epidemiology and pathophysiology of co-infection.

The PN panel can semiquantify the bacterial targets. In this study, of the 59 analytes with negative results by the culture methods, bacterial targets of 10^4–5^ copies/mL were detected in 36 analytes (61.0%) (Table 4). Quantitative values below the defined thresholds, such as 10^3^ CFU/mL for the protected specimen brush and 10^4^ CFU/mL for BAL, might be used as indicators for discontinuing antibiotics against ventilator-associated pneumonia (VAP) [3]. Therefore, the semiquantitative values determined by the PN panel may be useful for the management of VAP.

Meanwhile, we should consider the possibility of bacterial colonization, regardless of the detection method used. However, because *P. aeruginosa* and MRSA colonization are reported as the risk factors for subsequent infection [15, 16], detecting colonized bacteria would be helpful for prompt initiation of treatment when infection occurs.

Detection using culture methods depends on pathogen viability. Therefore, it is possible that the PN panel detected nonviable bacteria in this study, as one of the causes of discrepant results in bacterial detection between the PN panel and culture methods. There are other possibilities that the PN panel detected bacteria that were low-abundance or not cultured because of fastidious growth characteristics [8]. Therefore, the results of molecular assays should be carefully interpreted when used for patient management, in consideration of the differences in characteristics between molecular assays and culture methods.

Additionally, even if antimicrobial resistance markers are not detected, antimicrobial susceptibility can be decreased through other resistance mechanisms. Therefore, culture methods are necessary to test antimicrobial susceptibility. Further studies are required to establish practical strategies for complementary use of PN panel and traditional culture-based methods.

This study had some limitations. First, we did not collect all the samples submitted to our laboratory during the study period. Therefore, the data did not represent the epidemiology in our hospital. Second, we did not examine viral targets detected by the panel using a reference method and performed culture for MRSA using selective media only when requested by physicians. Third, the sample preparation process, including the sample volume, for the assay by the PN panel was off-label.

## Conclusions

The PN panel effectively detected a variety of pathogens in lower respiratory tract specimens. Further studies are needed to clarify its effects on patient management and cost-effectiveness.

## Data Availability

Not applicable.

## Abbreviations

LRIs: Lower respiratory tract infections
PN panel: BioFire FilmArray Pneumonia Panel
AST: Antimicrobial susceptibility testing
BAL: Bronchoalveolar lavage fluid
MRSA: Methicillin-resistant *Staphylococcus aureus*
MALDI-TOF MS: Matrix-assisted laser desorption ionization time-of-flight mass spectrometry
ESBL: Extended-spectrum β-lactamase
MIC: Minimal inhibitory concentration
SCC*mec*: Staphylococcal cassette chromosome *mec*
MREJ: *mec* right-extremity junction
PPA: Positive percent agreement
NPA: Negative percent agreement
VAP: Ventilator-associated pneumonia
